# Causal association between inflammatory cytokines and osteonecrosis: A bidirectional 2-sample Mendelian randomization study

**DOI:** 10.1097/MD.0000000000043671

**Published:** 2025-08-01

**Authors:** Wenkang You, Zhangdian Lin, Yanbin Lin, Mingzhong Liu, Rongjie Ye, Rongdong Zeng

**Affiliations:** a Quanzhou First Hospital of Fujian Medical University, Quanzhou, Fujian, China; b The School of Clinical Medicine, Fujian Medical University, Fuzhou, Fujian, China; c West China Xiamen Hospital of Sichuan University, Xiamen, Fujian, China.

**Keywords:** bidirectional Mendelian randomization, CDCP1, CSF1, inflammatory cytokines, LDSC, osteonecrosis

## Abstract

Increasing evidence suggests that immune-inflammatory responses are closely associated with osteonecrosis (ON). However, the specific inflammatory regulators involved in this pathogenesis remain unclear. Using a bidirectional Mendelian randomization (MR) investigation, we methodically investigated circulating inflammatory proteins that are causally linked to osteonecrosis. The genetic tools for 91 inflammatory circulating proteins were from a genome-wide association study with 14,824 participants, most of whom were from Europe. The ON summary statistics were acquired from the FinnGen database. The main strategy for drawing conclusions about causality was the inverse-variance weighted (IVW) method. The final results were supported by a number of sensitivity studies, including MR-Egger, weighted median, simple mode, weighted mode, and linkage disequilibrium score regression (LDSC). A link between CDCP1 and a higher risk of osteonecrosis has been revealed by genetic data (IVW OR = 1.23, 95% CI = 1.01–1.50, *P* = .037). Increased concentrations of CSF1 (IVW OR = 0.72, 95% CI = 0.54–0.96, *P* = .026), IL-10RB (IVW OR = 0.84, 95% CI = 0.699–0.999, *P* = .049), and MCP-4 (IVW OR = 0.81, 95% CI = 0.67–0.99, *P* = .038) were associated with a reduced risk of ON, while ON did not significantly affect these proteins. Only IL-18 may be a protective factor against osteonecrosis, according to reverse MR data (IVW OR = 0.97, 95% CI = 0.94–0.999, *P* = .042). This MR study investigated the causal associations between 91 inflammatory cytokines and osteonecrosis in detail overall. We discovered causal connections between reverse magnetic resonance imaging (reverse MR) and CDCP1, CSF1, MCP-4, and IL-18. These elements might contribute significantly to the pathophysiology of ON and might present fresh treatment options for the illness.

## 1. Introduction

Osteonecrosis (ON), also defined as avascular necrosis or aseptic necrosis, it pathogenesis has not been elucidated, is characterized as bone cell death that follows an impairment of the blood flow to the bone from a traumatic or non-traumatic origin.^[1,2]^ This devastating disease is an increasing worldwide health problem, particularly among young and middle-aged individuals, the increasing prevalence may be partly due to the use of more adjuvant therapies (corticosteroids, chemotherapeutic agents, and antiretroviral therapy for HIV-AIDS), as well as the increasing prevalence of many associated diseases and/or risk factors, and partly due to raised awareness of this condition.^[3,4]^ According to a study, the incidence rate of non-traumatic osteonecrosis of the femoral head was 2.51 cases per 100,000 person-years in the Japanese population (individuals between the ages of 40 and 59 years demonstrated relatively high incidence rates), In the United States, it is estimated that 20,000 to 30,000 new patients are diagnosed with osteonecrosis annually.^[5,6]^ Usually ON may be asymptomatic in the early stages, as the lesion develops, pain is the most common symptom in patients, such as necrosis of the femoral head often appears with groin pain and worsened during weight bearing or joint motion, eventually the joint will have severe mobility disorders, which seriously affects the quality of life.^[7–9]^ The occurrence of ON is related to many factors, for example, traumatism, dysbarism (or caisson disease), glucocorticoid use, alcoholism, sickle cell disease, systemic lupus erythematosus, hereditary thrombophilia/hypofibrinolysis^[10–14]^ In recent years, more and more studies have shown the close association between inflammatory cytokines and ON.^[15]^

Low molecular weight (≈6–70 kDa) soluble proteins called inflammatory cytokines are released by a range of cells, contributing to the immune response and offering important insights into the diagnosis, progression, and prognosis of different illnesses.^[16]^ Numerous inflammatory cytokines have been linked to ON, and each one plays a unique role.^[17]^ According to research by Wang et al, IL-34 can exacerbate steroid-induced ONFH by promoting osteoclast development through ERK, STAT3, and non-canonical NF-κB pathways. As a result, IL-34 may be a target for therapy.^[18]^ In Chinese patients, Jin et al investigated the relationships between polymorphisms of the IL-4 gene and steroid-induced ONFH. They discovered that the IL-4 gene may be linked to susceptibility to steroid-induced ONFH.^[19]^ Everything mentioned above raises the possibility that inflammatory cytokines and ON have a complicated relationship. Thus, using MR analysis, we examined the intricate causal relationship between 91 immunocyte cytokines and ON in this study. New concepts for investigating the mechanism, diagnosis, and therapy of ON will be generated by this work.

Using genetic diversity as a natural experiment, Mendelian randomization (MR) is a research strategy that provides evidence for the proposed causal links between diseases and modifiable risk variables.^[20]^ Because each allele has an equal chance of being inherited randomly by an individual, MR investigations are similar to randomized controlled trials in that they can help minimize biases from confounding and reverse causality.^[21,22]^ In the past, Lu et al discovered links between osteonecrosis, bFGF, IL-2, and IL-2RA.^[23]^ Instead, we explore inflammatory cytokines that may be causally linked to ON using a bigger genome-wide association study (GWAS) dataset using bidirectional 2-sample MR analysis. This could provide novel insights for early intervention, clinical management of ON, and preventative efforts in the future.

## 2. Methods

### 2.1. Study design

**Figure 1.**
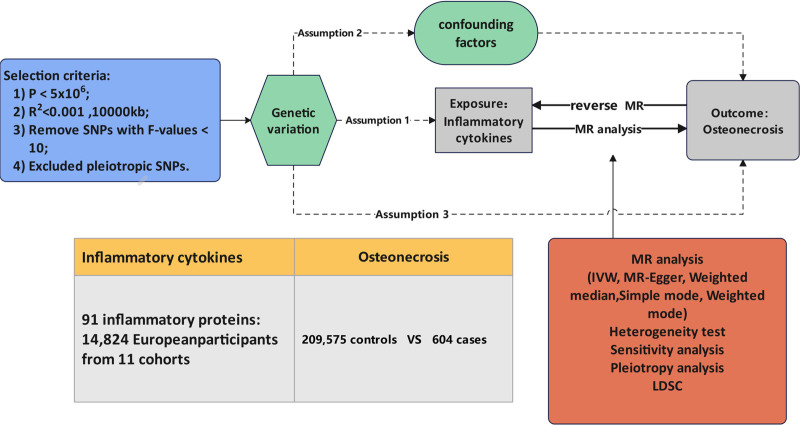
Study design diagram for MR analysis. We screened 91 inflammatory cytokines and instrumental variables for osteonecrosis, and then explored bidirectional causal relationships. MR = Mendelian randomization.

A bidirectional 2-sample MR analysis was used in this investigation to evaluate the causal connection between osteonecrosis and 91 inflammatory cytokines. Three fundamental presumptions must be met by the instrumental variable (IV) selection process in order to evaluate the causal relationship between exposure and outcome: It must satisfy the following 3 requirements: it must be consistently and substantially correlated with the exposure; it cannot be correlated with confounders, or variables that skew the relationship between exposure and outcome; and it is only correlated with the outcome as a result of the exposure (i.e., it is independent of the outcome given the exposure).^[24]^ The research design is shown in Figure 1.

### 2.2. Data sources

Our study follows the principle of MR, and the STROBE-MR checklist can be found in Supplementary Document. The FinnGen consortium (https://www.finngen.fi/en) conducted a publically available GWAS that included 604 cases and 209,575 controls. All of the populations involved in the GWAS were European. This is where the summary-level statistics for the associations with ON were collected.^[25]^ The GWAS catalogue (https://www.ebi.ac.uk/gwas/downloads) and the GWAS catalogue database (ID: GCST90274758–GCST90274848) provided the inflammatory cytokine data used in this work. Each inflammatory cytokine data set’s details are tabulated.^[26]^

### 2.3. Selection of IVs

When choosing the IVs, we followed the 3 fundamental presumptions mentioned above to guarantee effective IVs. To start, we used a significance threshold of *P* < 5E-06 to find the 91 inflammatory cytokines and the single nucleotide polymorphisms (SNPs) of the outcomes.^[27]^ Second, the parameter *r* threshold was set to 0.001 and the SNPs’ distance was set to 10,000 kb for the study in order to prevent LD of SNPs from influencing the results.^[28]^ Thirdly, we used the *F*-statistic to evaluate each SNP’s strength; SNPs with an *F*-statistic >10 were deemed to be substantially associated. In order to prevent any potential impact on the results, SNPs with a *F*-statistic of <10 were eliminated due to the likelihood of weak IV bias.^[29]^

### 2.4. Statistical analysis

Using the TwoSampleMR package, bidirectional 2-sample MR analyses were carried out in this work using R 4.3.2 software. Random effects and inverse-variance weighted (IVW) analysis were the primary techniques employed. Additional analysis techniques included MR-Egger, weighted median, simple mode, and weighted mode.^[30]^ The inverse of the ending variance, or the quadratic of se, is used as the weight for the fit in the IVW technique, which is distinguished by the fact that it ignores the existence of an intercept factor.^[31]^

In order to account for potential pleiotropy, we performed a number of sensitivity studies following the MR analysis, including the heterogeneity and pleiotropy tests. The IVs’ heterogeneity is measured using the Cochran *Q* test, where *P* < .05 denotes no heterogeneity. Pleiotropy is detected using MR-Egger regression, which also assesses its effect on the risk estimate of the intercept test when *P* > .05 denotes the absence of pleiotropy.^[32]^ Furthermore, the leave-one-out sensitivity test was employed to determine the potential substantial impact of a single SNP on the causative effect. Odds ratios (ORs) and 95% confidence intervals (CI) were used to determine causality, and a *P*-value of <0.05 indicated statistical significance.

To estimate genetic connection, LDSC regression analysis uses only GWAS summary statistics.^[33]^ It can correct for sample overlap, demographic stratification, and polygenicity-related bias. Using the R programme, we calculated LD scores to find the genetic associations between osteonecrosis and inflammatory cytokines. When *P* < .05, genetic link might be taken into account.^[34]^

## 3. Results

### 3.1. Influence of 91 inflammation cytokines on osteonecrosis

**Figure 2.**
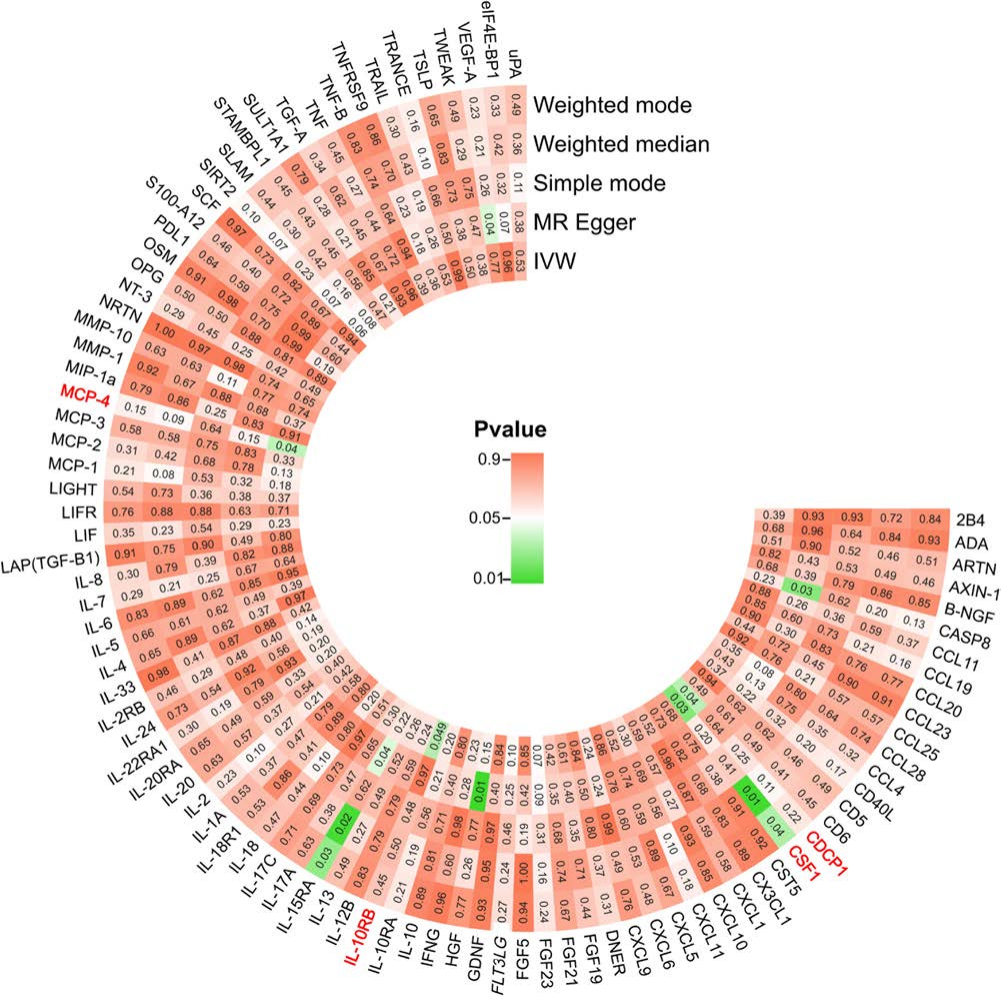
The heatmap displays *P*-values for various MR methods, including weighted mode, weighted median, simple mode, MR-Egger, and inverse-variance weighted (IVW), across the selected cytokines. Each cell represents the *P*-value for the causal effect of a specific cytokine on osteonecrosis, color-coded from green (*P* < .05) to red (*P* > .05), indicating the strength of evidence for a causal relationship. MR = Mendelian randomization.

**Figure 3.**
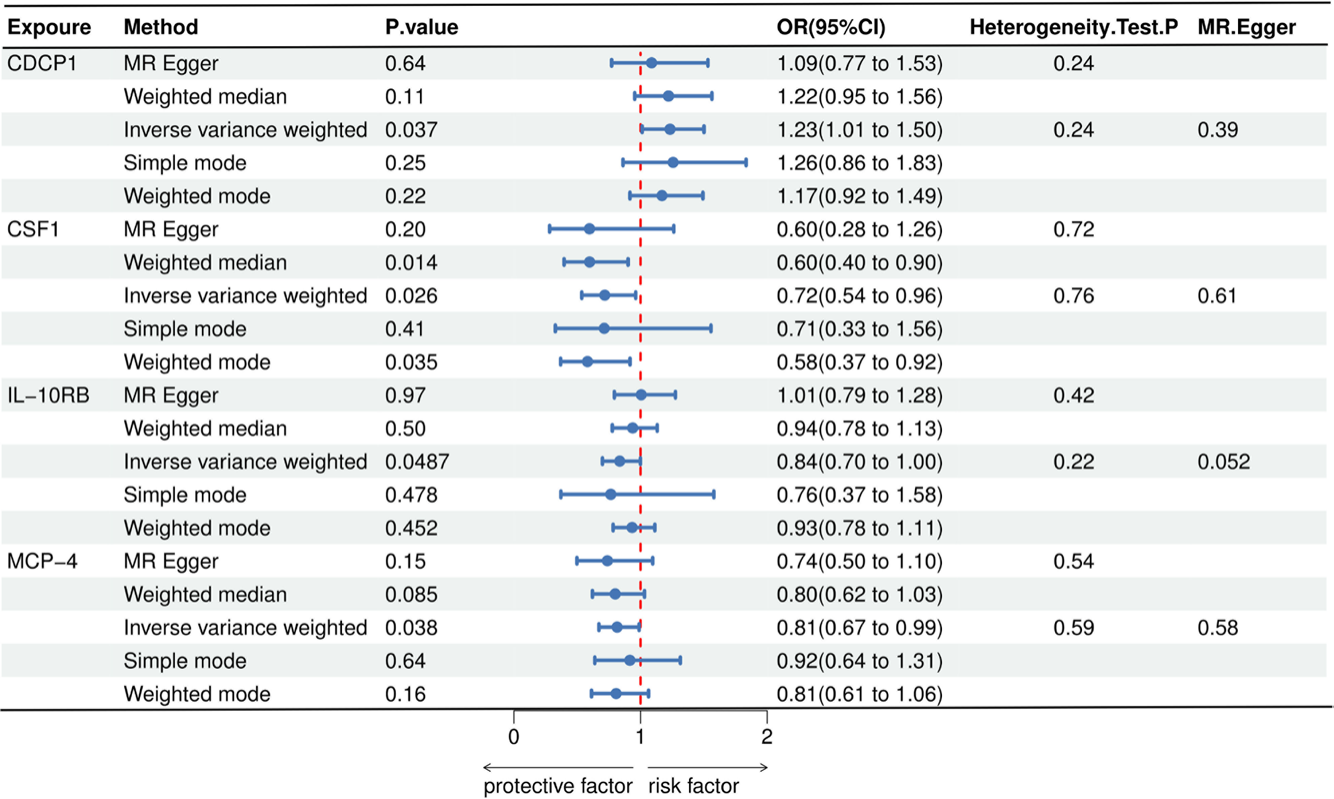
The forest plot visually represents the Odds ratios (OR) and their confidence intervals (CI) for each MR method across the 4 cytokines. The red dashed line at OR = 1 serves as the reference, indicating no effect. Values to the right of the line suggest a potential risk factor, while values to the left suggest a protective factor. MR = Mendelian randomization.

In this study, we screened the GWAS data of 91 inflammation cytokines for IVs (IVs), ensuring each cytokine had multiple SNPs as their IVs, all with *F*-values exceeding 10. As such, there was no weak IV bias in our study. Table S1 (Supplemental Digital Content, https://links.lww.com/MD/P573) provides a full description of the traits of the discovered SNPs. The IVW approach results showed that osteonecrosis was directly related to 4 inflammatory cytokines. Osteonecrosis and the CDCP1 cytokine showed a positive correlation, suggesting a causal relationship between CDCP1 and an elevated risk of osteonecrosis (IVW OR = 1.23, 95% CI = 1.01–1.50, *P* = .037). A persistent but non-significant link was revealed by the MR-Egger analysis (OR = 1.09, 95% CI = 0.77–1.53, *P* = .638). On the other hand, 3 cytokines showed a negative causal effect on osteonecrosis, suggesting a decreased risk: MCP-4 (IVW OR = 0.81, 95% CI = 0.67–0.99, *P* = .038), IL-10RB (IVW OR = 0.84, 95% CI = 0.699–0.999, *P* = .049), and CSF1 (IVW OR = 0.72, 95% CI = 0.54–0.96, *P* = .026). Consistent but non-significant trends were also discernible from the MR-Egger analysis (OR = 0.60, 95% CI = 0.28–1.26, *P* = .200; OR = 1.01, 95% CI = 0.79–1.28, *P* = .97; OR = 0.74, 95% CI = 0.50–1.10, *P* = .151). The results and additional analysis are depicted in Figures 2 and 3.

### 3.2. Forward sensitivity analysis

**Figure 4.**
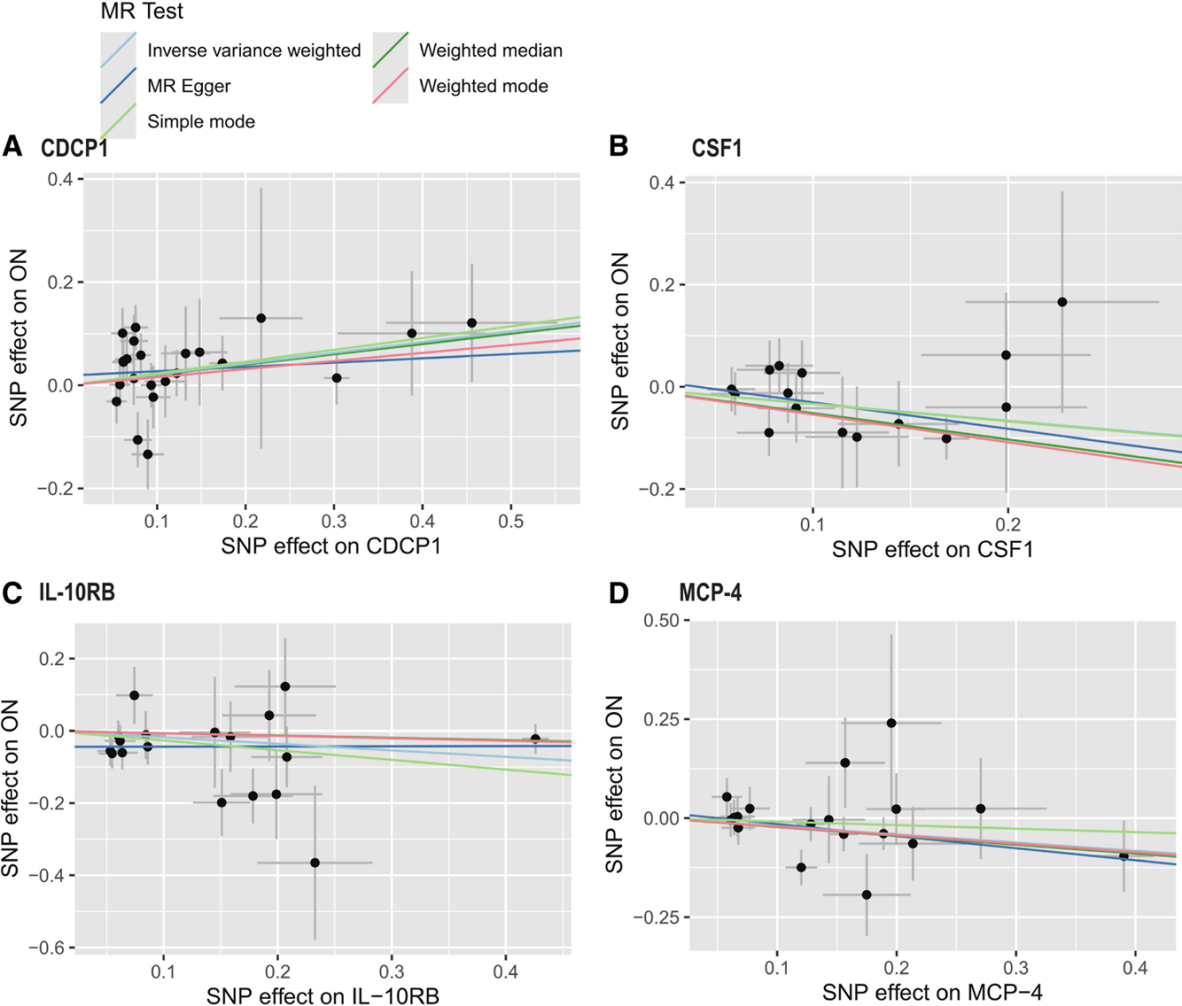
Scatter plot for causal effects of CDCP1, CSFI, IL-10RB, and MCP-4 on osteonecrosis, respectively. Analyses were performed by using the inverse variance weighted, MR-Egger, weighted median, weighted mode, and simple mode methods. The slope of each line corresponds to the estimated MR effect per method. Each Black point represents an SNP. MR = Mendelian randomization, ON = osteonecrosis, SNP = single nucleotide polymorphism.

Based on the IVW and MR-Egger analyses in the Cochran *Q* test (fig or table), the results of the sensitivity analyses demonstrated that none of the 4 inflammatory cytokines for MR analysis of osteonecrosis were heterogeneous (*P* > .05); nor were they horizontally pleiotropic (MR-Egger intercept method *P* > .05), demonstrating the credibility of the causally robust results (Fig. 3). Figure 4 and Figure S1 (Supplemental Digital Content, https://links.lww.com/MD/P572) show the scatter plot and leave-one-out analysis results obtained from the MR analysis. The leave-one-out analysis indicates that the causal relationship between IL-10RB and osteonecrosis is driven by influential SNPs, suggesting that the findings should be carefully considered and conclusions should be interpreted cautiously. In contrast, the causal relationships between CDCP1, CSF1, MCP-4, and osteonecrosis are not driven by influential SNPs, indicating that our conclusions are robust (Fig. S1, Supplemental Digital Content, https://links.lww.com/MD/P572).

### 3.3. LDSC regression analysis

**Figure 5.**
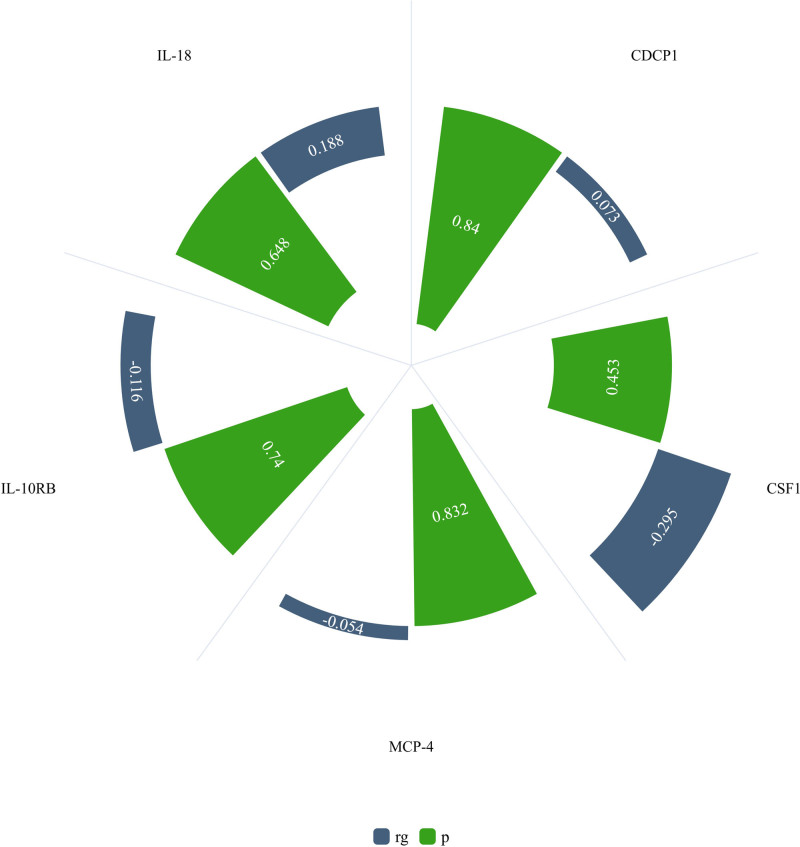
Nightingale rose diagram of genetic correlation between CDCP1, CSF1, MCP-4, LI-10RB, IL-18 and osteonecrosis. p = significance of genetic correlations, Rg = magnitude of the genetic correlation.

We analyzed the genetic correlation between these inflammatory cytokines and osteonecrosis. We do not observe the existence of substantial genetic correlations (rg = 0.073 and *P* = .840 between CDCP1 and osteonecrosis; rg = -0.295 and *P* = .453 between CSF1 and osteonecrosis; rg = -0.054 and *P* = .832 between MCP-4 and osteonecrosis; rg = -0.116 and *P* = .740 between IL-10RB and osteonecrosis; and rg = 0.188 and *P* = .648 between osteonecrosis and IL-18). These results suggests that our conclusions are not affected by coheritability (Fig. 5).

### 3.4. Causal effects of osteonecrosis on inflammatory proteins

Based on the IVW technique results, one inflammatory cytokine has a causal relationship with osteonecrosis. An association between the inflammatory cytokine IL-18 and osteonecrosis is found to be negative, indicating that IL-18 may operate as a protective factor against osteonecrosis (IVW OR = 0.97, 95% CI = 0.935–0.999, *P* = .042) Table S2 (Supplemental Digital Content, https://links.lww.com/MD/P573) and Figure S2 (Supplemental Digital Content, https://links.lww.com/MD/P572) display the reverse MR analysis results.

### 3.5. Reverse sensitivity analysis

**Figure 6.**
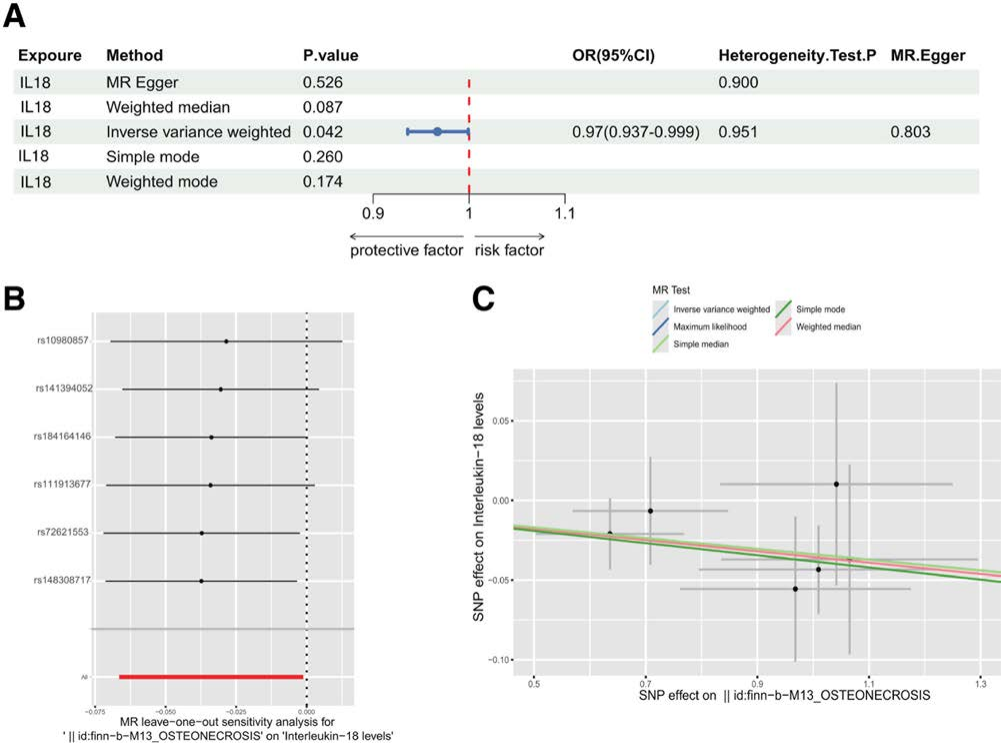
Causal effects of osteonecrosis on IL-18. (A) Forest plot for causal effects of osteonecrosis on IL-18. Horizontal bars indicate 95% confidence intervals. (B) “Leave-one-out” plots for the causal association between osteonecrosis and IL-18. (C) Scatter plot for causal effects of osteonecrosis on IL-18. CI = confidence interval, OR = odds ratio, SNP = single nucleotide polymorphism.

According to the IVW and MR-Egger analyses in the Cochran *Q* test, the results of the sensitivity analyses indicated that there was neither heterogeneity nor horizontal pleiotropy in the MR analysis of IL-18 on osteonecrosis (*P* > .05); this demonstrated the credibility of the causally robust results (Fig. 6A). Figure 6B and C displays the scatter plot and leave-one-out results from the MR analysis. Plots from the leave-one-out analysis showed that there was no potentially significant SNP driving the causal relationship between osteonecrosis and IL-18, supporting our finding that the relationship was stable.

## 4. Discussion

In this investigation, we used a bidirectional 2-sample MR approach to thoroughly assess the causal connections between osteonecrosis and 91 inflammatory cytokines. The forward MR analysis showed a correlation between a higher incidence of osteonecrosis and elevated levels of inflammatory cytokines. On the other hand, data from the reverse magnetic resonance study revealed a possible link between altered levels of inflammatory cytokines and an elevated risk of osteonecrosis. These results further complicate the link between inflammatory proteins and osteonecrosis by suggesting a possible bidirectional causal relationship between the 2.

According to our findings, osteonecrosis is directly associated with 5 inflammatory cytokines. To be more precise, there was a negative correlation found between the risk of osteonecrosis and CSF1, LI-10RB, and MCP-4, and a positive correlation with CDCP1. Additionally, an increased risk of osteonecrosis may lead to decreased levels of IL-18. These findings were robust in sensitivity analyses and were not influenced by pleiotropy or confounding factors. The identification of these inflammatory cytokines could contribute to the early diagnosis, treatment, and prevention of osteonecrosis.

Osteonecrosis has numerous etiologies and pathophysiological mechanisms, which remain incompletely understood. In recent years, the emerging field of osteoimmunology has elucidated the close relationship between inflammatory cytokines and osteonecrosis.^[30,32,35]^ For instance, some studies have shown that TNFα inhibits osteogenesis and further damages bone tissue, while MCP-1 can induce monocyte/macrophage proliferation and promote osteoclast differentiation and activation.^[36,37]^ Previous research has indicated that overexpression of CDCP1 in extracellular vesicles, in the presence of NF-κB ligand, facilitates osteoclastogenesis,^[38]^ suggesting its involvement in the development of osteonecrosis. This aligns with our study findings.

Emoto et al found that CSF-1 regulates osteoclastogenesis mediated by osteoblasts and plays a significant role in bone development.^[39]^ Furthermore, marrow adipogenic lineage precursors are the main source of CSF1 in bone and one of the main cell types driving bone resorption, according to research by Zhong et al osteosclerosis and osteocyte abnormalities result from the inhibition of bone resorption caused by a CSF1 deficit.^[40]^ Current research on the relationship between CSF1 and osteonecrosis is limited; our study demonstrates a negative causal association between CSF1 and osteonecrosis. According to research by Cavalcante et al, LI-10RB has anti-inflammatory properties, and its decreased expression can lead to weakened anti-inflammatory responses and increased pro-inflammatory mediators.^[41]^ Our findings open up new avenues for further investigation by confirming a negative causal relationship between IL-10RB and osteonecrosis. In rheumatoid arthritis, MCP-4 is a monocyte chemoattractant protein that is extensively expressed. It causes joint damage by promoting the proliferation of rheumatoid synovial cells. It is also upregulated in osteoarthritis and can serve as an inflammatory marker of the disease.^[42,43]^ Thus, the relationship between MCP-4 and osteonecrosis warrants further investigation based on our research findings. Additionally, IL-18 has anti-inflammatory properties and may have a protective role against bone erosion by inhibiting osteoclast formation.^[44]^ Additionally, a negative connection between IL-18bp and bisphosphonate-related osteonecrosis of the jaw was shown by de Barros Silva et al. One possible treatment target for osteonecrosis could be IL-18.^[45]^

Notably, studies using MR have been conducted before ours to examine the connection between inflammatory cytokines and osteonecrosis.^[23]^ However, our research differs in several key aspects. Firstly, the datasets used in our study are different, with our dataset being larger and encompassing a greater number of inflammatory cytokines. Secondly, we employed a bidirectional 2-sample MR analysis method. Additionally, we conducted LDSC regression analysis to exclude the influence of genetic correlation on the experimental results. Lastly, our conclusions differ, as we identified entirely different causal associations between inflammatory cytokines and osteonecrosis. This study serves as a supplement and enhancement to their research. To completely understand the connection between different inflammatory cytokines and osteonecrosis, more research is required.

Our study does, however, have a few drawbacks. First off, this study’s data came only from European sources and was limited to populations in Europe. We have not validated these results in other populations, and our study only considers population-level effects without accounting for individual differences. Secondly, to evaluate heterogeneity and pleiotropy, we used MR-Egger regression and Cochran *Q* test. While these methods can statistically mitigate the impact of heterogeneity and pleiotropy, they cannot entirely eliminate their influence in a clinical context. Moreover, our research is subject to the same restrictions as MR analyses, including the incapacity to conduct stratified analyses according to characteristics like age and gender. Finally, by offering suggestions for next research, this work advances the investigation of the causal link between inflammatory cytokines and osteonecrosis through the use of MR techniques. Nonetheless, more experimental validation is required before any clinical inferences can be made.

## 5. Conclusions

In summary, our research suggests that inflammatory variables play a major role in the development of osteonecrosis and may offer fresh perspectives on how to manage this illness.

## Acknowledgments

We are grateful to all those who participated in or contributed to this research project. We would like to extend our sincere thanks to the members of our research team for their dedication and hard work. We also appreciate the public data provided by the Finnish database and the research data on inflammatory factors by Zhao et al.

## Author contributions

**Data curation:** Wenkang You, Rongjie Ye.

**Formal analysis:** Zhangdian Lin.

**Funding acquisition:** Rongdong Zeng.

**Methodology:** Rongdong Zeng.

**Project administration:** Rongdong Zeng.

**Resources:** Yanbin Lin, Rongdong Zeng.

**Software:** Wenkang You.

**Supervision:** Mingzhong Liu, Rongdong Zeng.

**Visualization:** Wenkang You, Zhangdian Lin, Rongdong Zeng.

**Validation:** Yanbin Lin, Mingzhong Liu, Rongdong Zeng.

**Writing – original draft:** Wenkang You, Zhangdian Lin.

**Writing – review & editing:** Wenkang You, Zhangdian Lin, Rongjie Ye.

## Supplementary Material


