# Supplementary material for: Causal association between inflammatory cytokines and osteonecrosis: A bidirectional 2-sample Mendelian randomization study
Source: Medicine (Baltimore). 2025 Aug 1;104(31):e43671. doi: 10.1097/MD.0000000000043671 (PMC12323912; doi:10.1097/MD.0000000000043671)

**Figure S1:** Leave-one-out analysis for CDCP1, CSF1, IL-10RB, and MCP-4 showing the effect of removing each SNP on the causal estimates for osteonecrosis.


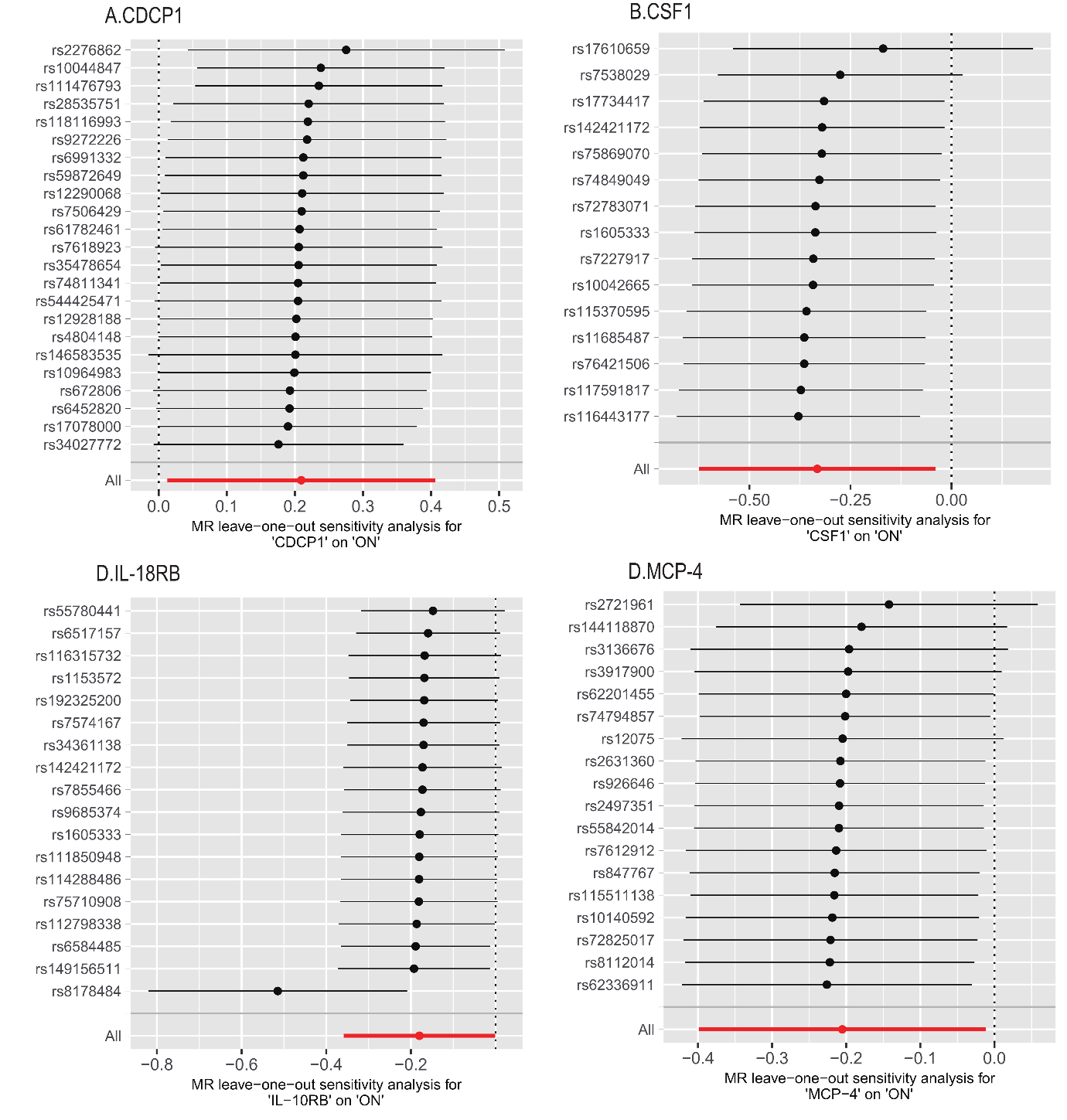


**Figure S2:** Funnel plots for CDCP1, CSF1, IL-10RB, and MCP-4 showing the distribution of SNP-level effects in the MR analysis for osteonecrosis.


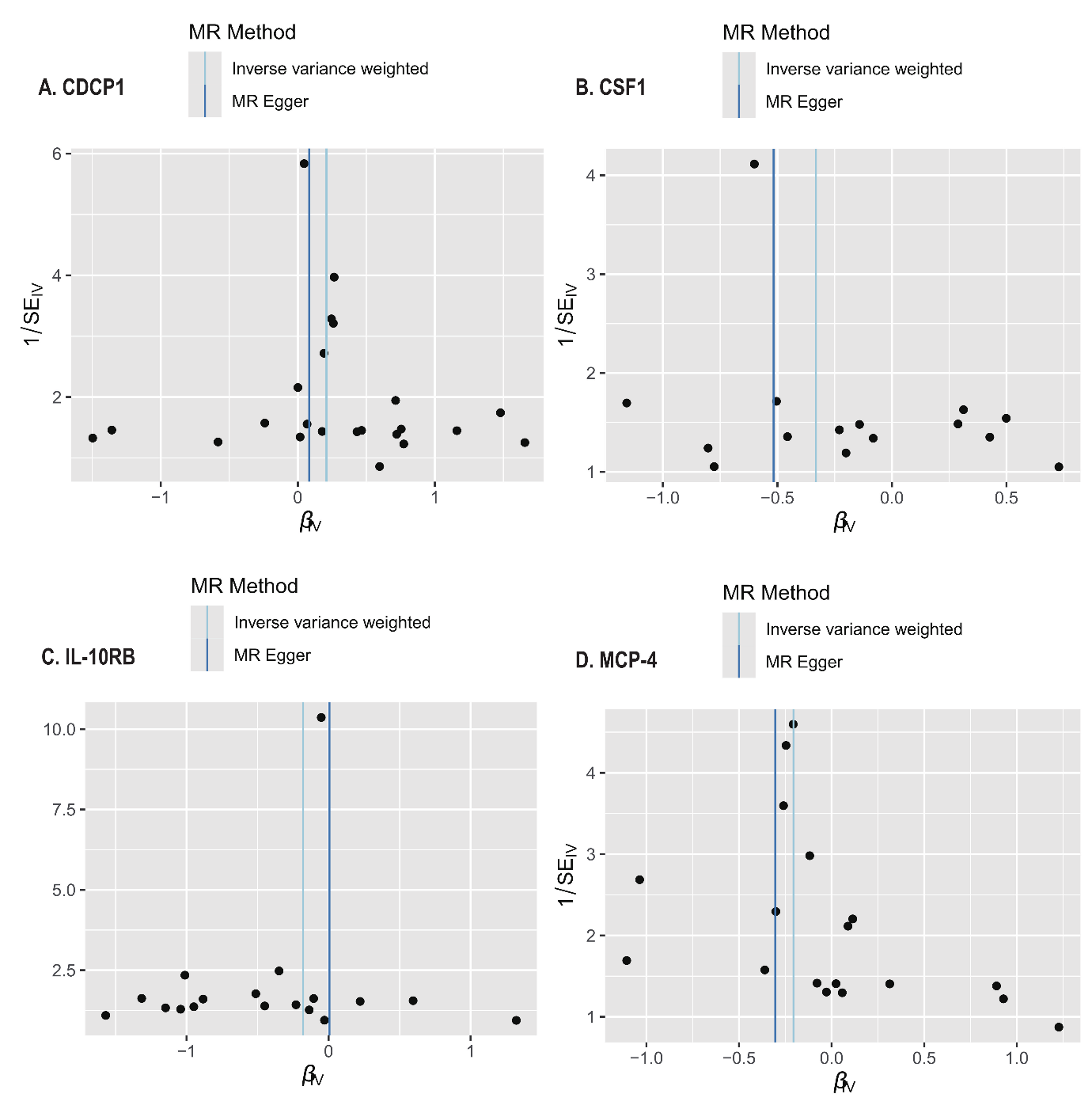

Supplement: Supplementary file 2 [file medi-104-e43671-s002.docx]
